# A Compressive Peak Strength Model for CFRP-Confined Thermal Insulation Materials under Elevated Temperature

**DOI:** 10.3390/ma13010026

**Published:** 2019-12-19

**Authors:** Yeou-Fong Li, Wai-Keong Sio, Ying-Kuan Tsai

**Affiliations:** 1 Department of Civil Engineering, National Taipei University of Technology, 1, Section 3, Chung-Hsiao E. Rd., Taipei 10608, Taiwan; yfli@ntut.edu.tw (Y.-F.L.); qoo147896325@gmail.com (W.-K.S.); 2 Department of Environmental Information and Engineering, Chung Cheng Institute of Technology, National Defense University, 75, Shiyuan Rd., Daxi Dist., Taoyuan 335, Taiwan

**Keywords:** peak strength, carbon fiber-reinforced polymer, thermal insulation, perlite, elevated temperature

## Abstract

In this paper, a compressive peak strength model for CFRP-confined thermal insulation materials under elevated temperature was proposed. The thermal insulation material was made by Portland cement with different portions of perlite. The compressive strengths of four different perlite ratios in weight, such as 0%, 10%, 20%, and 30% of thermal insulation materials, confined by one-layer, two-layer, and three-layer carbon fiber-reinforced polymer (CFRP) composite materials, were obtained. The test results indicated that the specimen’s compressive strength decreased with an increase in the amount of perlite replacement and increased with an increase in the number of CFRP wrapping layers. Based on the test results, a theoretical compressive peak strength model with some parameters was proposed. In the meantime, the compressive strengths of the above four different perlite ratios of thermal insulation materials under elevated temperature, such as ambient temperature, 100 °C, 150 °C, 200 °C, 250 °C, and 300 °C, were obtained. For compression tests of specimens with a fixed amount of perlite, the test results indicated that the specimen’s compressive strength decreased with an increase in temperature, highlighting a thermal softening phenomenon. Based on the test results, a compressive peak strength model with a thermal softening parameter was proposed to predict the peak strength under elevated temperature. Finally, a compressive peak strength model for thermal insulation material with CFRP confinement under different elevated temperature was derived, and it achieved acceptable results in comparison to the experimental results.

## 1. Introduction

**Figure 1:**
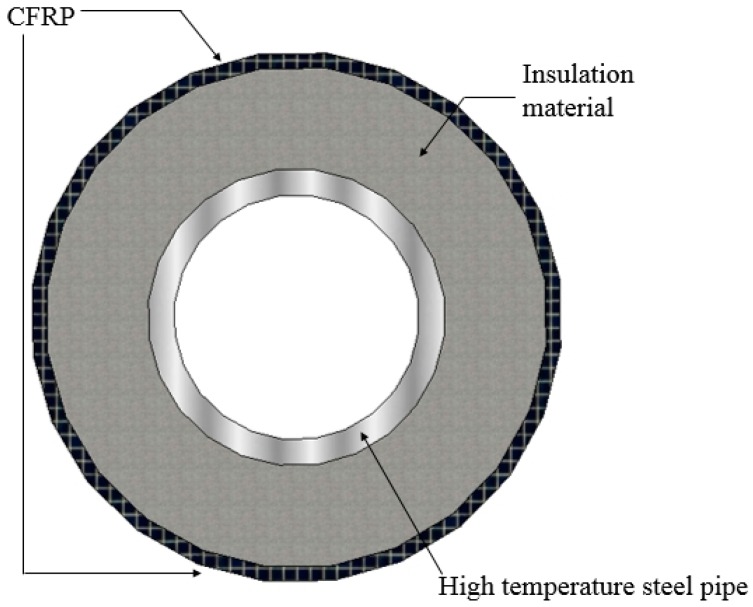
The strengthening method for high-temperature steel pipe.

As a global industry, the petrochemical industry is an inextricable part of our lives as it is a vital material for consumers and high-tech products. Transmission mild steel pipelines are viewed as the most cost-efficient way to transport petroleum products. However, these pipelines are sensitive to corrosion in harsh environments, particularly in the presence of acid, chloride, and sulfur ingress media. The common solution for repairing a damaged steel pipe is to cover it with a patch made of carbon fiber or mineral wool. The current strengthening methods for coating thermal insulation materials on the surface of pipelines still have issues to overcome, especially when the corroded pipeline is installed in a high-temperature environment. A strengthening method for high-temperature steel pipelines was proposed by Li et al. [1], where inorganic insulation materials confined by CFRP composite materials were used to strengthen the damaged pipeline under elevated temperature, as shown in Figure 1. To improve the performance of the strengthening method discussed in Reference [1] by mixing with other potential additives, we investigate the effect of elevated temperature and perlite replacement ratio on the performance of CFRP-confined concrete under compression. In addition, a compressive peak strength model for CFRP-confined thermal insulation materials under elevated temperature is proposed.

The advantages of CFRP applications include high strength, anti-corrosion, light weight, and ease of construction. However, it cannot maintain its mechanical properties at high temperatures. Thus, a composite material made from Portland cement, perlite, and CFRP confinement was proposed as a strong and durable solution for repairing metal pipelines. The experimental studies were carried out by conducting compressive strength tests on the specimens made of Portland cement with different ratios of perlite added and under different temperatures. In addition, the CFRP-confined and unconfined specimens were made for compression tests. This study aims at developing a compressive peak strength model for CFRP-confined perlite insulating concrete under elevated temperature. A number of studies were reviewed, and they are summarized below.

The performance of concrete under compression in high-temperature conditions shows that the strength of concrete decreases with an increase in temperature. Calcined perlite powder was added into the concrete for a compression test, and it showed that the compressive strength decreased with the increase in perlite replacement ratio. The thermal conductivity and mechanical performance of lightweight concrete showed that the compressive strength decreased with an increase in the amount of lightweight aggregate [2,3,4]. The ultra-lightweight cement composite was exposed to high-temperature and showed that elastic modulus loss was significantly quicker than that of compressive strength. Increasing the expanded perlite powder in the lightweight concrete showed that the compressive strength and elastic modulus decreased [5,6].

Some compressive peak strength models were proposed for predicting the peak strength of concrete confined by steel reinforcement and FRP composite materials [7,8,9,10,11]. The behavior of FRP-confined concrete and unconfined specimens exposed to elevated temperatures resulted in concrete losses as the rate of temperature increased. The properties affecting the behavior of FRP-confined concrete and the confinement effectiveness decreased with an increase in the compressive strength of concrete [12,13,14].

Perlite is a construction material used for heat insulation. Upon adding different percentages of perlite, the compressive strength decreased with an increase in the perlite ratio of the heat insulation materials [15,16]. The compressive strength of the concrete increased upon the replacement of expanded perlite and pumice aggregates. By adding bottom ash in the lightweight concrete, the compressive strength and thermal conductivity increased, while it decreased upon adding aluminum. The material density and compressive strength decreased following the introduction of expanded perlite aggregates, pumice aggregates, and rubber aggregates into the concrete [17,18,19].

The CFRP and fibre reinforced cementitious matrix (FRCM) elements at elevated temperatures in the climatic chamber and the load-bearing capacity gradually decreased with an increase in temperature [20,21]. For the compressive behavior of concrete cylinders confined by CFRP composite material, the compressive strength increased upon using CFRP composite jackets. The compressive strength of a square reinforced concrete column increased by wrapping it with CFRP composite materials. The CFRP-enclosed concrete cylinders had an obvious size effect under varying CFRP confinement ratios, and the degree of CFRP confinement could significantly improve the strength [22,23,24,25].

## 2. Experimental Program

In this paper, the experimental program involved the concrete mix test and compressive strength test to investigate the impact of high temperature on the efficiency of externally confined thermal insulation material with CFRP sheets. Thus, the peak strength for different numbers of wrapping layers of CFRP under certain elevated temperatures was proposed.

### 2.1. Materials

The materials used in this research included Portland cement, expanded perlite, carbon-fiber sheets, and epoxy. Portland cement is a general-purpose cement suitable for all uses; it is composed of calcium, silicon, aluminum, iron, and small amounts of other compounds. Due to its characteristics of high hydration heat, low early strength, and long curing time, it is generally used in infrastructure construction.

**Figure 2:**
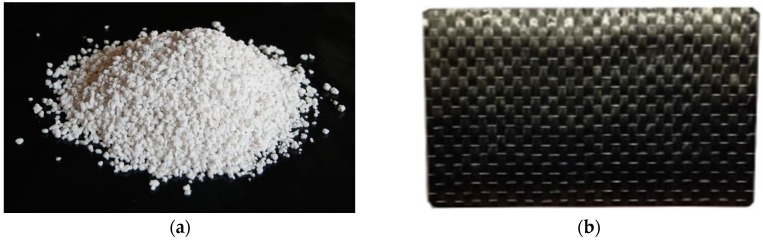
Expanded perlite powder (**a**) and carbon fiber-reinforced polymer (CFRP) (**b**).

Expanded perlite is an amorphous volcanic glass, and it is composed of about 70% silicon dioxide and 14% alumina. When it is heated above 870 °C, the volume increases to 4–20 times its original volume and becomes porous, which is called expanded perlite. Its characteristic of porosity makes it a good insulation material, and it is widely used in building construction, agriculture, and chemical engineering fields. The grain size of the expanded perlite powder used in this study ranged from 1 mm to 6 mm, as shown in Figure 2a.

CFRP is made of a polymer matrix reinforced with carbon fibers, and it is a composite material. Recently, carbon fiber was widely used in automotive, aerospace, and civil engineering applications. The CFRP composite material has features of acid and alkali resistance, anti-corrosion, and a high strength-to-weight ratio [26]. The material properties of the carbon-fiber sheet and epoxy resin are shown in Table 1. The CFRP sheet used in the present study was a uni-directional one to provide better confinement performance, as shown in Figure 2b.

### 2.2. Samples and Testing Procedure

This study conducted cylindrical specimen compressive testing under ambient temperature in accordance with the ASTM C39/C39M-18 [27], which entailed placing specimens in a universal testing machine, with each specimen at a loading rate of 900–1800 N/s (strain rate of 10^−6^/s to 10^−4^/s) to obtain the maximum compressive strength at its seven-day curing age. This test program was undertaken by the 100-ton force universal testing machine at the material laboratory of the Department of Civil Engineering, National Taipei University of Technology.

In addition, the perlite powders were added to Portland cement with four perlite ratios based on weight (0%, 10%, 20%, and 30%), and the water–cement ratio for Portland cement was 0.4. Then, the compressive strength of cubic specimens was obtained under different temperatures (ambient temperature, 100 °C, 150 °C, 200 °C, 250 °C, and 300 °C) at the 28-day curing age with the same controlled loading rate. The specimens were placed in a crucible and heated in a furnace, and then they were removed from the furnace for compressive strength test immediately after reaching the required temperature. The compressive strength tests of standard cubic specimens with dimensional aspects of 5 cm × 5 cm × 5 cm were performed according to ASTM C109/C M109-02 [28]. Table 2 gives the name of the specimens and testing methods, and Table 3 lists the mix design proportions for insulating material specimens. In Table 2, CPC stands for the cement with perlite specimen for compression test, CPCC stands for the cement with perlite specimen confined by CFRP composite material for compression test, and CPTC stands for the cement with perlite specimen under elevated temperature for compression test.

The other measuring apparatus employed in this study included an infrared thermometer (measurement range from −35 °C~550 °C), a strain gauge (elongation limit up to 2%), and a data acquisition system. The identification and the number of cylinders and cubic specimens are listed in Table 4 and Table 5, respectively.

Forty-eight cylindrical specimens (10 cm in diameter and 20 cm in height) were tested to investigate the effect of CFRP wrapping on the compressive strength of insulation materials. The unconfined and CFRP-confined specimens (one, two, and three layers of CFRP) were tested considering four different ratios of perlite replacement based on weight (0%, 10%, 20%, and 30%).

**Figure 3:**
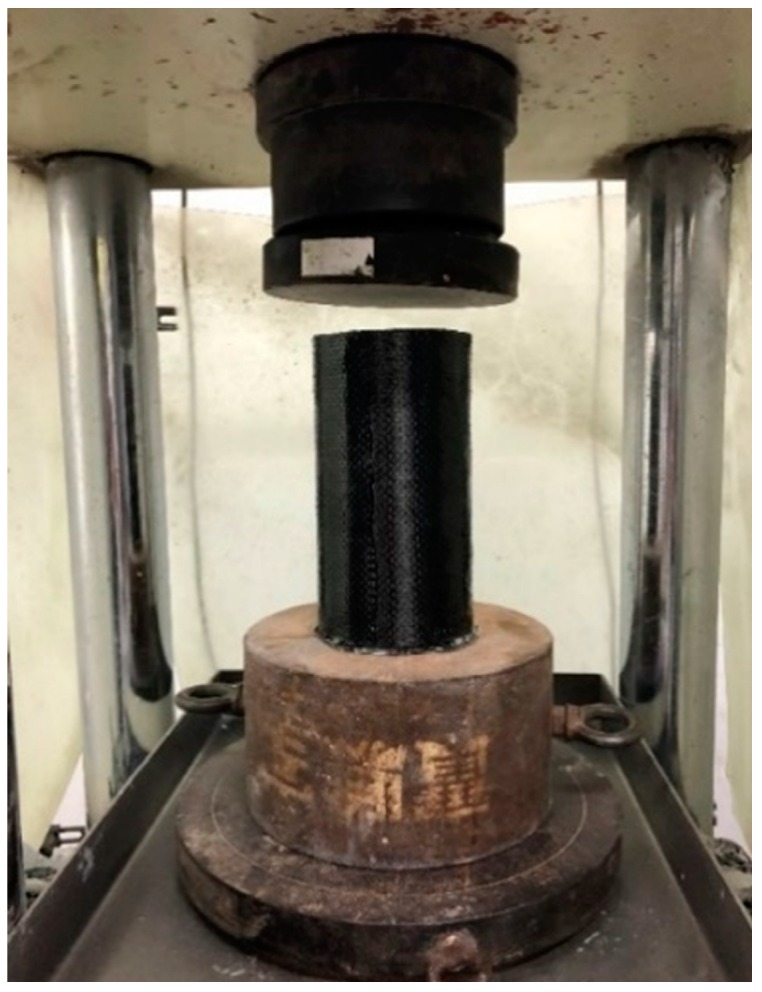
Compression test for cylindrical concrete confined by CFRP composite material.

The applied procedures of CFRP attachment are described below. The surface was cleaned before applying CFRP layers with an epoxy-based coating to ensure a good bond between the outer surface of the cylinders and CFRP. A thin layer of primer epoxy was firstly applied to the surface of the cylinders. After the primer epoxy was cured at ambient temperature for several hours, the carbon-fiber sheet was applied to the cylinders. For each layer of carbon-fiber sheet, the epoxy was applied using a paintbrush to fully saturate the carbon fiber. After the required sheet layers were applied, the CFRP jacketing was cured at ambient temperature. The length of the overlay was more than 10 cm, and the duration of applying the next layer was more than one day. The CFRP tension strain was measured by circumferential strain gauges glued to the middle surface of the CFRP-wrapped specimens, and the elongation limit of the strain gauges was as high as 2%. The compression test for a concrete cylinder confined by CFRP composite material is shown in Figure 3.

## 3. Compression Test of the Insulation Material Confined by CFRP

The compression test results of the insulation material without/with CFRP are described in the subsections below. The experiment aimed at investigating the effect of perlite additions (ratios in weight: 0%, 10%, 20%, and 30%) on the compressive strength of the specimens. 

### 3.1. Compression Test on Specimens without CFRP Wrapping

**Figure 4:**
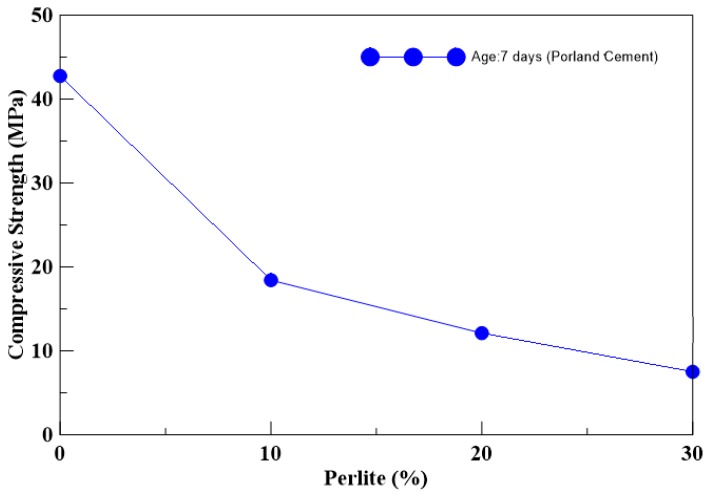
Compressive strength of the cylindrical specimens with different perlite ratios.

**Figure 5:**
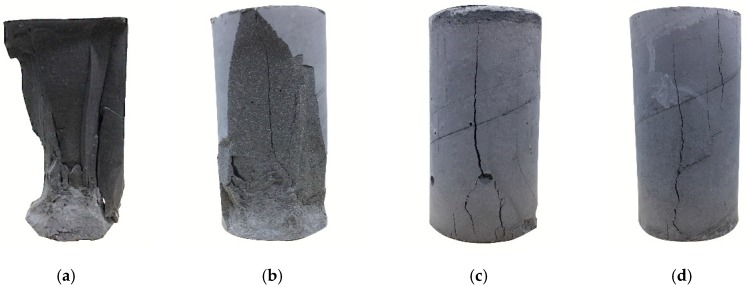
Failure modes of the unconfined cylindrical specimens after compression test, (**a**) CPC0; (**b**) CPC10; (**c**) CPC20; (**d**) CPC30.

A series of compression tests on the unconfined cylindrical specimens were carried out to observe the change in strength with different mix designs. Table 6 shows the test results, and the results showed that the compressive strength decreased with an increase in perlite addition. As seen from Table 6, the strength of Portland specimens with 10% perlite was less than half that of the pure cement specimen. The compressive strengths of the cylindrical unconfined specimens changed according to different perlite content, as shown in Figure 4. Figure 5 shows the failure modes of the specimens after testing. For lower perlite ratios of the specimen, the failure mode was a brittle failure.

### 3.2. Compression Test on CFRP-Confined Specimens

The compression test on the CFRP-confined specimens with four perlite ratios in weight (0%, 10%, 20%, and 30%) was conducted to observe the effect of the number of wrapping layers.

#### 3.2.1. Portland Cement with 0% Perlite 

**Figure 6:**
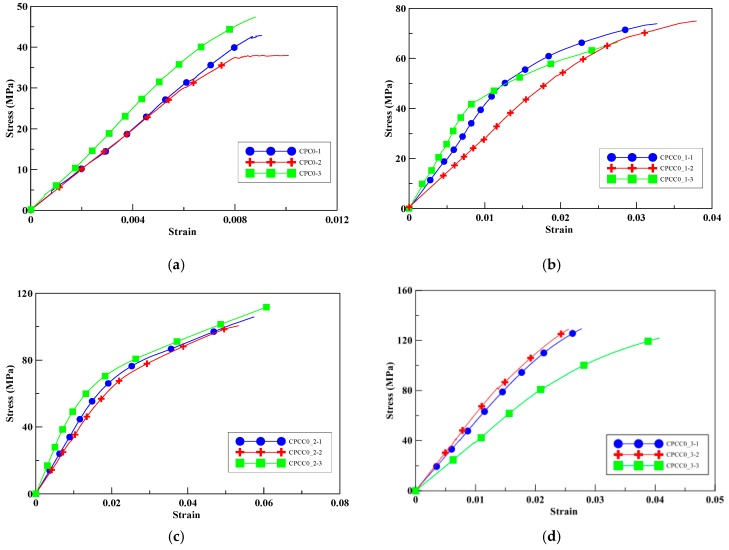
Experimental stress–strain curves for test specimens with 0% perlite, (**a**) unconfined; (**b**) one-layer CFRP; (**c**) two-layer CFRP; (**d**) three-layer CFRP.

To study the effect of perlite addition on the compressive peak strength of Portland cement cylinders wrapped with different numbers if CFRP layers, a compression test on specimens with 0% perlite addition was conducted as a control group. Figure 6a illustrates the axial stress versus the axial strains for the unwrapped cylinders; the average ultimate strength was 42.75 MPa. As seen in Figure 6b–d, the compressive peak strengths of the specimens CPCC0_1, CPCC0_2, and CPCC0_3 were increased by 67–202% with an increase in the number of CFRP wrapping layers.

Moreover, the ultimate strain of CPCC0_1 and CPCC0_2 was increased by 259–520%. These improvements illustrate the confinement effect on the compressive strength enhanced by CFRP. The ultimate strain improvement of CPCC0_3 was less than that of CPCC0_2, which may be attributed to the stronger confinement strength that constrained the deformation before failure, leading to brittle failure. In CPCC0_2, it should be noted that an abrupt drop in strength occurred when loaded at about 60 MPa due to part of the CFRP composites rupturing; the strength was regained until a full rupture of CFRP occurred. Table 7 shows the fractured specimens at failure, and Table 8 shows the average compressive peak strengths and their corresponding increase percentages.

#### 3.2.2. Portland Cement with 10% Perlite

**Figure 7:**
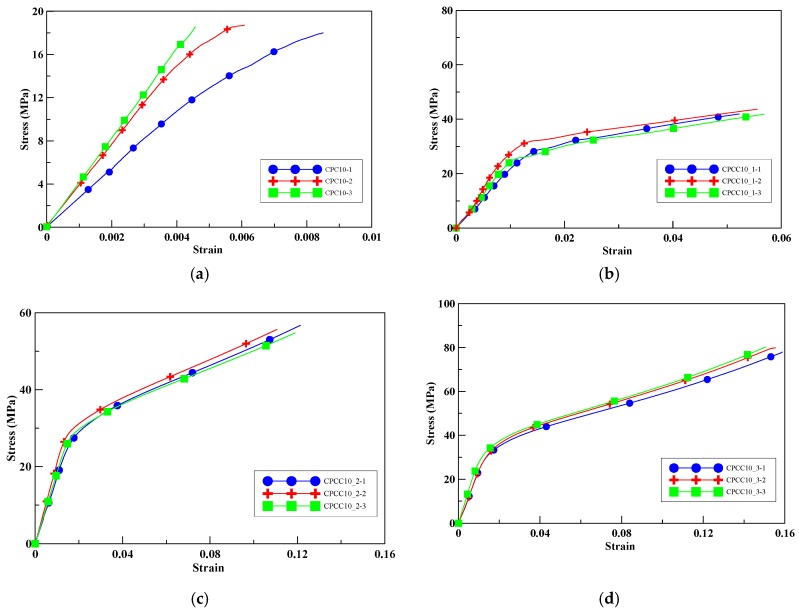
Experimental stress–strain curves for test specimens with 10% perlite, (**a**) unconfined; (**b**) one-layer CFRP; (**c**) two-layer CFRP; (**d**) three-layer CFRP.

The average compressive peak strength of the unwrapped cylinders with a 10% perlite additive was 18.42 MPa, as shown in Figure 7a. With an increase in the number of CFRP wrapping layers, the compressive strength of the specimens CPCC10_1, CPCC10_2, and CPCC10_3 was increased by 131–331%, as shown in Figure 7b–d. The confinement effect on compressive peak strength improvement was more significant than for the specimens without perlite additive.

#### 3.2.3. Portland Cement with 20% Perlite

**Figure 8:**
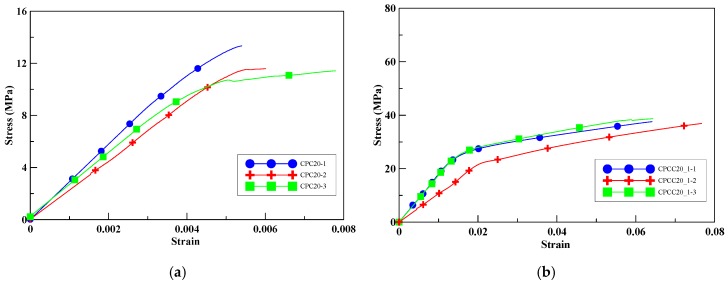

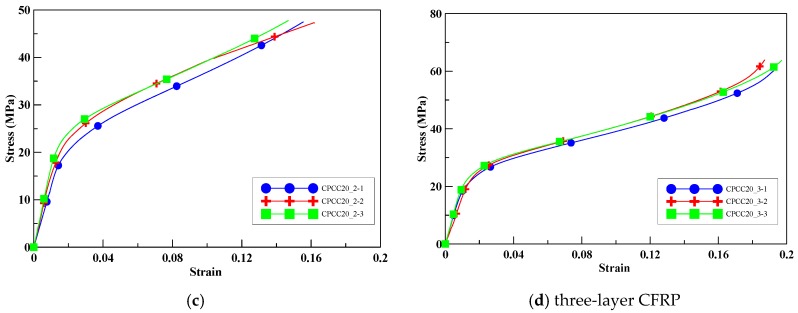
Experimental stress–strain curves for test specimens with 20% perlite, (**a**) unconfined; (**b**) one-layer CFRP; (**c**) two-layer CFRP; (**d**) three-layer CFRP.

With 20% perlite replacement, the compressive strength of the unwrapped specimens was decreased by 71.67% compared to the unconfined specimens without CFRP, as shown in Figure 8a. As seen from Figure 8b–d, the compressive peak strengths of the specimens CPCC20_1, CPCC20_2, and CPCC20_3 were increased by 211–416% with an increase in the number of CFRP wrapping layers. It is again illustrated that, at a given ratio of perlite addition, the compressive peak strength increased with the number of CFRP wrapping layers.

#### 3.2.4. Portland Cement with 30% Perlite

**Figure 9:**
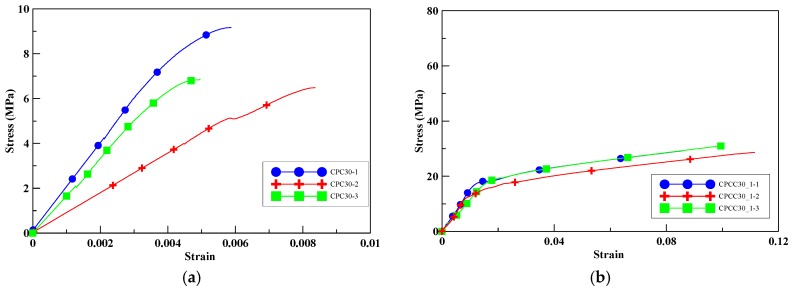

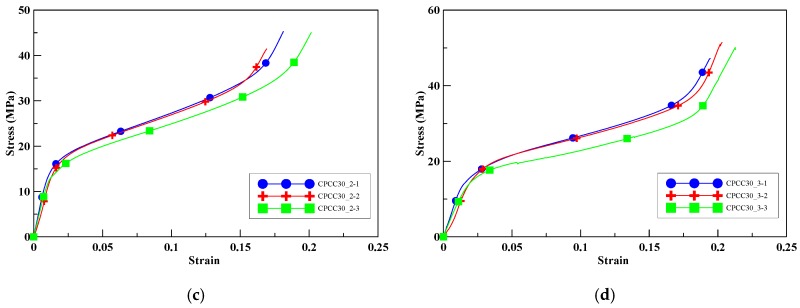
Experimental stress–strain curves for test specimens with 30% perlite, (**a**) unconfined; (**b**) one-layer CFRP; (**c**) two-layer CFRP; (**d**) three-layer CFRP.

When the perlite additive increased to 30%, the ultimate strength of the unconfined cylinders decreased to 7.5 MPa, as shown in Figure 9a. With an increase in the perlite addition, the compressive strength decreased. With an increase in the number of CFRP confined layers, the compressive strength of the specimens CPCC30_1, CPCC30_2, and CPCC30_3 was increased by 296–559%, as shown in Figure 9b–d. The improvement percentage of the specimens was the most significant among the four groups of specimens with different amounts of perlite additives. This trend illustrates that the compressive strength enhanced by CFRP was more effective in specimens with relatively low strength. It should be noted that, in CPCC30_3, a strength loss could be observed on the stress–strain curves at about 10 MPa, indicating the damage of the CFRP composite. It reached the peak strength when the CFRP completely ruptured and failed to carry the extra load. Moreover, the hoop tension provided by three layers of CFRP wrapping was stronger than the compressive strength of the core part of the specimens. Thus, it allowed the specimens to withstand more load. It was observed that, for all CFRP-confined specimens, the failure was due to the rupture of CFRP composite materials, accompanied by a loud sound.

### 3.3. Discussion on CFRP-Confined Specimen Compression Test Results

From the above test results, the compressive peak strength of the insulation material specimens increased due to confinement by different numbers of layers of CFRP composite material, as compared to those specimens without confinement. Table 8 shows the compressive peak strengths of the insulation material specimens and their increase percentages. As the number of layers of CFRP wrapping increased, so did the percentage of compressive peak strength.

## 4. Compressive Test Results of the Insulation Material at Elevated Temperature

**Figure 10:**
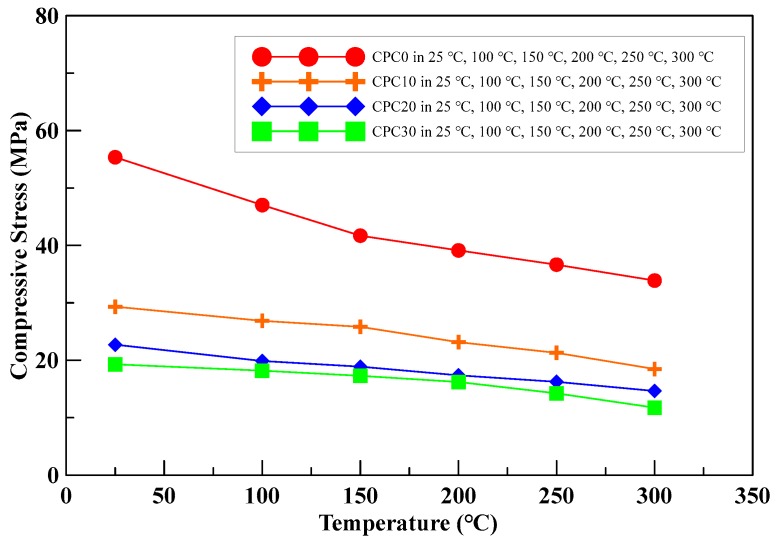
Compressive strength of the insulating material specimens with various perlite ratios and temperatures.

This experiment aimed at investigating the effect of perlite addition (ratios in weight: 0%, 10%, 20%, and 30%) on the residual compressive strength of the insulating material specimens under different temperatures (ambient temperature = 25 °C, 100 °C, 150 °C, 200 °C, 250 °C, and 300 °C). The compressive strength test was applied to standard cubic specimens with dimensional aspects of 5 cm × 5 cm × 5 cm. A series of compression tests on cubic specimens with different mix designs under elevated temperatures were conducted to observe the changes in strength after 28-day curing age. The specimens were placed in a crucible and heated, and then they were removed from the furnace to cool down; when they reached the required temperature, the compressive strength tests were conducted immediately. The peak strength decreased with an increase in temperature. The compressive stress–temperature relationships of the specimens are shown in Figure 10. As shown in Table 9, the results indicated that the insulating material specimens’ compressive peak strength decreased with an increase in perlite addition.

The comparison results indicated that the ratio of perlite in weight plays a significant role in the efficiency of insulation material specimens under elevated temperatures. For compression tests of specimens under a given temperature, higher ratios of perlite addition in weight led to lower compressive strength. Moreover, for compression tests of specimens with a fixed amount of perlite, a higher temperature led to a lower compressive strength.

## 5. Compressive Peak Strength Model

This study developed a peak strength model suitable to represent the compressive behavior of insulation material specimens under different temperatures confined with different numbers of CFRP wrapping layers using regression analysis of the test data. The thermal softening parameter of the peak strength was obtained from the experimental results. The peak strength of the insulation materials confined by CFRP was derived from the Mohr–Columb failure envelope theory, which can be explicitly expressed as a function of the strength of unconfined insulation material, the lateral confining stress, and the angle of internal friction of insulation material. A combined peak strength model of thermal insulation material with CFRP confinement under elevated temperature is proposed. Comparing the peak strengths of the proposed model with that of the experimental results, it was found that the proposed model can predict the peak strength of the perlite insulation material with acceptable accuracy. It is noted that the CFRP-confined specimens were tested at room temperature, and the influence of the temperature on CFRP properties was not taken into account. The derivation and analysis procedure is described below.

### 5.1. A Peak Strength Model for CFRP-Confined Insulation Material 

To investigate the effect of CFRP confinement on the strength of insulation material, the Portland cement-based specimens with perlite added were considered as an insulation material, and a series of compressive tests were conducted to observed the changes in strength due to the numbers of CFRP wrapping layers. A theoretical peak strength model for CFRP-confined concretes was proposed by Li et al. [7]. The peak strength model is expressed as follows:

(1) $${f^{\prime }}_{cc}={f^{\prime }}_{c}+{f^{\prime }}_{l}tan^{2}\left(45{}^{\circ}+\frac{\emptyset }{2}\right),$$

where

(2) $${f^{\prime }}_{l}=\frac{2\;\times \;n\;\times \;t\;\times \;E_{cf}\;\times \;\varepsilon_{cf}\;\times \;k_{c}}{D},$$

(3) $$\emptyset =36{}^{\circ}+1{}^{\circ}\left(\frac{{f^{\prime }}_{c}}{35}\right)\leq 45{}^{\circ}.$$

In Equation (1), *f’_cc_* and *f’_c_* are the confined and unconfined concretes for the compressive strength, respectively, *f’_l_* stands for the effective lateral confined stress of CFRP, and *ϕ* is the internal friction angle of concretes. In Equation (2), *n* is the number of layers of CFRP, *t* is the thickness of the single CFRP layer, *E_cf_* is the elastic modulus of CFRP, *ε_cf_* is the strain of CFRP measured at the CFRP-confined concretes, and *k_c_* is a sectional shape factor.

To investigate the performance of CFRP confinement, the effective lateral confined stress of CFRP was obtained using Equation (2). For one layer, two layers, and three layers of CFRP wrapping, the effective lateral confined stresses (*f’_l_*) were 7.84 MPa, 15.68 MPa, and 23.51 MPa, respectively. The measured strains of CFRP-confined specimens are listed in Table 10; we set *ε_cf_* = 1.0% in the calculation.

The perlite was added to the cement-based specimens as a porous material, which led to a lower strength than that of normal concretes; thus, the internal friction angle needed to be modified. Equation (3) was then modified as follows:

(4) $$\emptyset =A{}^{\circ}+B{}^{\circ}\times \left(\frac{{f^{\prime }}_{c}}{{f^{\prime }}_{l}}\right)\leq 45{}^{\circ},$$

where *f’_c_/f’_l_* and *ϕ* are used as the horizontal and vertical axes of coordinates; the theoretical angle of internal friction can then be obtained utilizing a regressive analysis method. Subsequently, the theoretical compressive peak strength of the confined specimens was obtained by substituting the internal friction angle (*ϕ*) into Equation (1).

**Figure 11:**
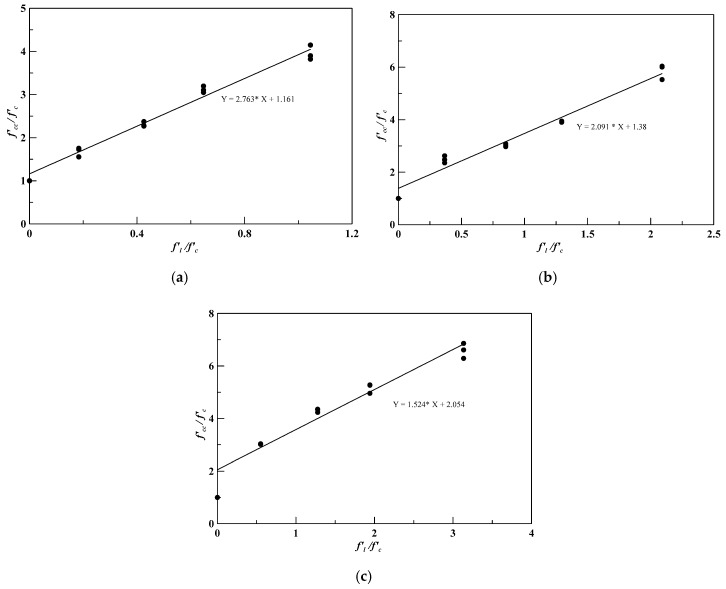
Regression analysis of the experimental data for determination of internal friction angle of CFRP-confined specimens, (**a**) one-layer CFRP; (**b**) two-layer CFRP; (**c**) three-layer CFRP.

To find the angle of internal friction, Equation (1) was modified as follows:

(5) $$\frac{{f^{\prime }}_{cc}}{{f^{\prime }}_{c}}=1+\frac{{f^{\prime }}_{l}}{{f^{\prime }}_{c}}\times tan^{2}\left(45{}^{\circ}+\frac{\emptyset }{2}\right),$$

where *f’_l_/f’_c_* and *f’_cc_/f’_c_* are used as the horizontal and vertical axes of coordinates shown in Figure 11, and then the internal friction angle can be obtained utilizing the regression analysis method. Figure 11a shows the results of the insulation material specimens wrapped by one-layer CFRP. From the regression analysis result, the experimental value of the internal friction angle was 27.9°. Figure 11b,c show the regression analysis results and indicate that the friction angles of the specimens wrapped by two-layer and three-layer CFRP were 20.7° and 12.0°, respectively. The internal friction angles of the model are shown in Table 11.

In Equation (4), *f’_c_/f’_l_* values were obtained from Table 12, and *ϕ* values are shown in Table 11, which were then used as the horizontal and vertical axes of coordinates. By using the regression analysis, the coefficients *A* and *B* in Equation (4) could be obtained as 16.46 and 2.37, respectively. Equation (4) was rewritten as follows:

(6) $$\emptyset =16.46{}^{\circ}+2.37{}^{\circ}\left(\frac{{f^{\prime }}_{c}}{{f^{\prime }}_{l}}\right)\leq 45{}^{\circ}.$$

**Figure 12:**
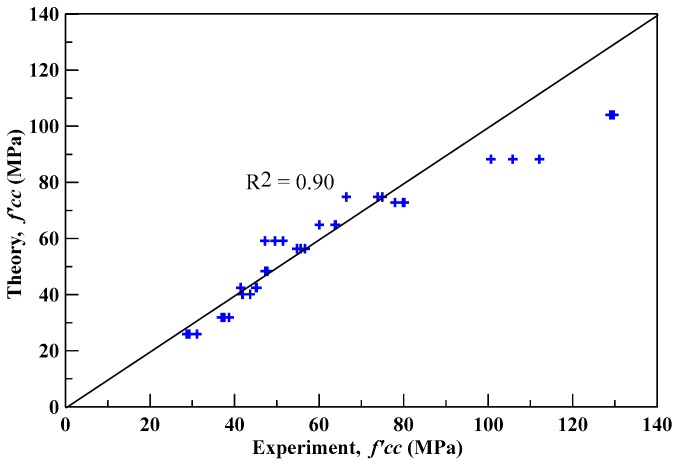
The deviation of theoretical and experimental compressive strengths of confined specimens.

The theoretical compressive peak strengths of specimens confined by CFRP were obtained by substituting the corresponding internal friction angle into Equation (1). Table 13 lists the experimental and the proposed theoretical compressive peak strengths and their percentage errors, where the average absolute error was found to be 9.48%. Figure 12 shows the deviation between the theoretical and experimental compressive peak strengths, and the correlation coefficient (*R^2^*) was equal to 0.9. The results show that the proposed theoretical compressive peak strength model could predict the experimental compressive peak strength with good accuracy.

### 5.2. A Peak Strength Model for Insulation Material under Elevated Temperature

The proposed peak strength model for cement-based insulation materials under elevated temperature is provided below.

(7) $${f^{\prime }}_{T}={f^{\prime }}_{c}\times e^{-\lambda \left(T-T_{ref}\right)},$$

where *f’_T_* is the compressive peak strength of the insulation material under elevated temperature (*T*), *f’_c_* is the compressive peak strength at reference temperature (*T_ref_*), where *T_ref_* is the reference (room) temperature, and λ is the thermal softening parameter of the insulation materials with different perlite ratios.

**Figure 13:**
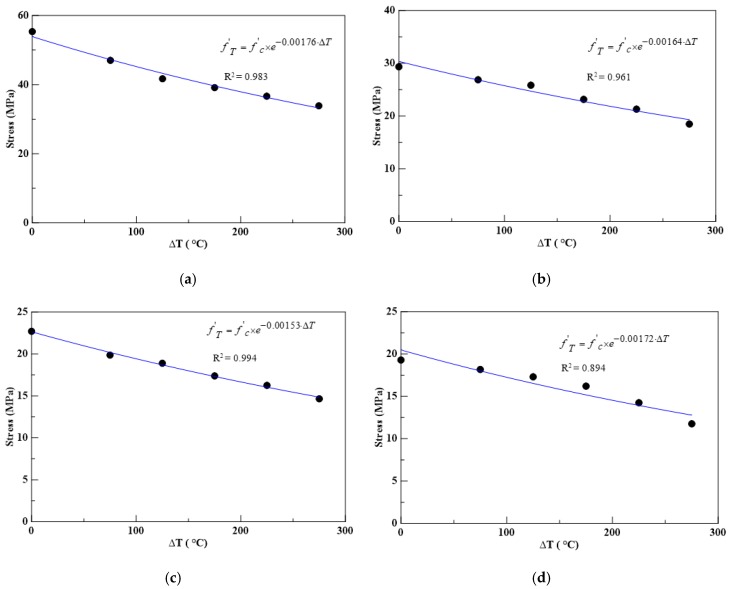
The regression analysis results of the material parameter of CPC with 0%, 10%, 20%, and 30% perlite, (**a**) 0% perlite; (**b**) 10% perlite; (**c**) 20% perlite; (**d**) 30% perlite.

The thermal softening parameter for each insulation material with different perlite ratios was determined by conducting a regression analysis on the compressive peak strength under different elevated temperatures. For the material parameters for partial replacement of cement with expanded perlite, the results of the regression analysis are shown in Figure 13, where *f’_c_* and Δ*T* = *T* − *T_ref_* were set as the horizontal and vertical axes of coordinates. The thermal softening parameters of the insulation materials with different perlite ratios are shown in Table 14.

**Figure 14:**
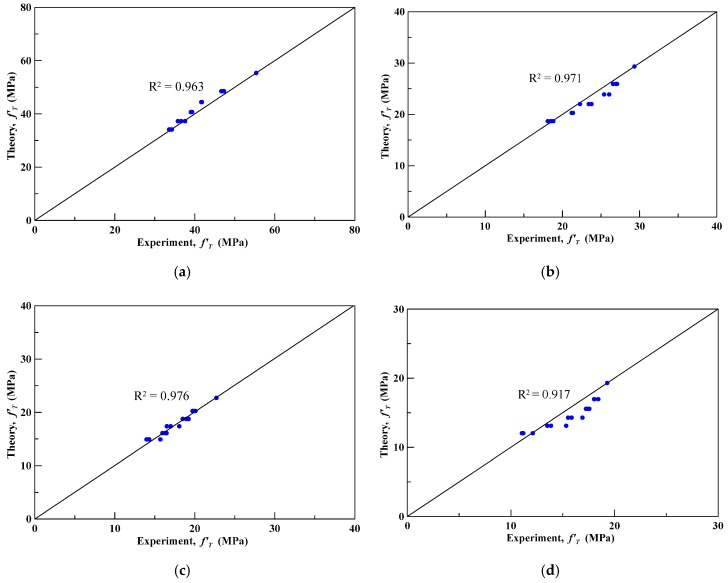
The deviation of theoretical and experimental compressive strengths of OPC specimens with 0%, 10%, 20%, and 30% perlite under elevated temperature, (**a**) 0% perlite; (**b**) 10% perlite; (**c**) 20% perlite; (**d**) 30% perlite.

Subsequently, the obtained λ in Table 14 was substituted into Equation (7) to compute the theoretical compressive peak strength of the insulation materials with different perlite ratios in weight. Table 15 lists the absolute errors between the theoretical and experimental compressive peak strengths; the average absolute errors were 2.81%, 3.71%, 2.37%, and 6.95% for perlite ratios of 0%, 10%, 20%, and 30%, respectively. Figure 14 shows the deviation of the theoretical and experimental compressive peak strengths, and all the correlation coefficients (*R^2^*) were between 0.92 and 0.98. The results show that the proposed theoretical compressive peak strength model, shown in Equation (7), could predict the experimental compressive peak strength with good accuracy.

### 5.3. A Peak Strength Model for CFRP-Confined Insulation Material under Elevated Temperature

For the peak strength model for CFRP-confined insulation material, the average absolute error between the theoretical and experimental peak strength results was 9.48%, and the correlation coefficient of experimental and theoretical results was 0.9. For the peak strength model for insulation material under elevated temperature, the average absolute errors between the theoretical and experimental peak strength results were between 2.37% and 6.95%, and the correlation coefficients (*R^2^*) were between 0.92 and 0.98. Thus, a peak strength model of CFRP-confined specimens under elevated temperature was developed by combining the compressive strength characteristics of the unwrapped specimens under elevated temperature and of the CFRP-wrapped specimens at room temperature. The physical-based peak strength model for CFRP-confined insulation material under elevated temperature is provided as follows:

(8) $${f^{\prime }}_{Tcc}={f^{\prime }}_{cc}\times e^{-\lambda \left(T-T_{ref}\right)},$$

where *f’_Tcc_* is the compressive strength of CFRP-confined specimens under elevated temperatures, *f’_cc_* is the compressive strength of CFRP-confined specimens shown in Equation (1), and λ is the thermal softening parameter. Equation (8) provides an alternative approach for prediction of the compressive strength of CFRP-confined specimens under elevated temperatures.

## 6. Conclusions

Regression analysis was used for predicting the compressive strength of thermal insulation specimens with and without CFRP wrapping using three variables, namely, the cement–perlite ratio in weight, elevated temperatures of the concrete, and the number of CFRP confined layers. Based on the results of this study, the following conclusions can be drawn:

1. From the test results, it is found that the compressive strength of Portland cement diminished with an increase in the addition of perlite.
2. In the compression tests under elevated temperatures, it was observed that the compressive strength of the insulation material specimens decreased with an increase in temperature.
3. In the compression tests of CFRP-confined specimens, it was found that a lower original compressive strength led to a higher strength enhancement percentage after the application of CFRP confinement. For the insulation material specimens with one-layer CFRP wrapping, the strength was 3.96 times higher than for unwrapped specimens. For the two-layer and three-layer CFRP-confined specimens, the compressive strength was 5.86 and 6.65 times higher than for unwrapped specimens. A higher number of layers of CFRP wrapping led to a higher percentage of compressive peak strength.
4. For the peak strength model for CFRP-confined insulation material, the average absolute error between the theoretical and experimental peak strength results was 9.48%, and the correlation coefficient of experimental and theoretical results was 0.9.
5. For the peak strength model for insulation material under elevated temperature, the average absolute errors between the theoretical and experimental peak strength results were between 2.37% and 6.95%, and the correlation coefficients (*R^2^*) were between 0.92 and 0.98.

## Figures and Tables

**Table 1:** Material properties of the carbon-fiber sheet and epoxy resin.

**Carbon-Fiber Sheet**	**Specification**	**FAW 300 (g/m^2^)**
	Young’s modulus, *E_cf_* (GPa)	250
	Tensile strength (GPa)	4.9
	Thickness (mm/layer)	0.16
	Ultimate strain	0.02
**Epoxy Resin**	Viscosity (cps)	1823 (at 25 °C)
	Young’s modulus (GPa)	3.5
	Tensile strength (MPa)	52.2
	Tensile adhesive strength (MPa)	10.5

**Table 2:** Identification of unconfined specimens. CFRP—carbon fiber-reinforced polymer.

Specimen	Description (Test Method)	Dimension	Perlite Ratio in Weight (%)
CPC	Cement with perlite (ASTM C39/C39M-18)	*Φ*10 cm × 20 cm	0; 10; 20; 30
CPCC	Cement with perlite confined by CFRP (ASTM C39/C39M-18)	*Φ*10 cm × 20 cm	
CPTC	Cement with perlite under elevated temperature (ASTM C109/C M109-02)	5 cm × 5 cm × 5 cm	

**Table 3:** Applied mix design for insulating material specimens.

Specimen	Perlite (g)	Perlite Ratio % (in Weight)	Portland Cement (g)	Water (g)
CPC0	0	0	2000	800
CPC10	200	10	1800	720
CPC20	400	20	1600	640
CPC30	600	30	1400	560

**Table 4:** Number of cylindrical specimens with different numbers of CFRP layers.

Specimen	Shape	Perlite Ratio in Weight (%)	No. of CFRP Layers	No. of Cylindrical Specimens
CPC0	Cylinder	0	0, 1, 2, 3	12
CPC10		10		12
CPC20		20		12
CPC30		30		12

Total number of specimens: 48.

**Table 5:** Number of cubic specimens under elevated temperatures.

Specimen	Shape	Perlite Ratio in Weight (%)	Elevated Temperatures (°C)	No. of CFRP Cubic Specimen
CPTC0	Cube	0	25, 100, 150, 200, 250, 300	12
CPTC10		10		12
CPTC20		20		12
CTPC30		30		12

Total number of specimens: 48.

**Table 6:** Compression test results of CFRP-confined specimens with perlite addition.

Specimen	Perlite Ratio in Weight (%)	Average Compressive Strength (MPa)	Decrease Percentage (%)
CPC0	0	42.75	-
CPC10	10	18.42	56.91
CPC20	20	12.11	71.67
CPC30	30	7.50	82.46

**Table 7:** Appearance of the CFRP-confined specimens after testing.

Specimen with Perlite Percentage (%)	1-Layer CFRP	2-Layer CFRP	3-Layer CFRP
0% perlite (CPC0)	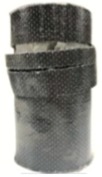	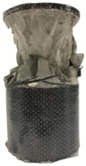	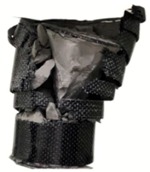
10% perlite (CPC10)	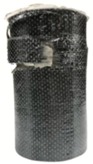	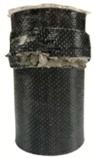	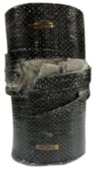
20% perlite (CPC20)	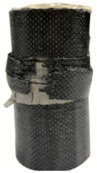	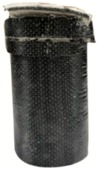	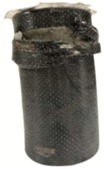
30% perlite (CPC30)	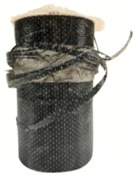	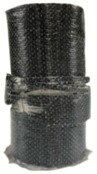	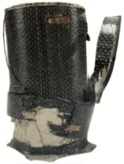

**Table 8:** Compression test results of specimens with perlite addition confined by different numbers of layers of CFRP.

Specimen	Perlite Ratio in Weight (%)	No. of CFRP Layers	Average Compressive Peak Strength (MPa)	Increase Percentage (%)
CPC0	0	-	42.75	-
CPCC0_1		1	71.76	67.4
CPCC0_2		2	106.18	148.4
CPCC0_3		3	129.32	202.5
CPC10	10	-	18.42	-
CPCC10_1		1	42.48	130.6
CPCC10_2		2	55.71	202.4
CPCC10_3		3	79.33	330.7
CPC20	20	-	12.11	-
CPCC20_1		1	37.73	211.6
CPCC20_2		2	47.51	292.3
CPCC20_3		3	62.58	416.8
CPC30	30	-	7.50	-
CPCC30_1		1	29.67	295.6
CPCC30_2		2	43.92	485.6
CPCC30_3		3	49.40	558.7

**Table 9:** Results of unconfined specimens at different elevated temperatures.

Specimen	Temperature	Perlite Ratio in Weight	Average Compressive Peak Strength (MPa)
CPT25C0	25 °C (room temperature)	0%	55.25
CPT25C10		10%	29.33
CPT25C20		20%	22.70
CPT25C30		30%	19.29
CPT100C0	100 °C	0%	47.02
CPT100C10		10%	26.82
CPT100C20		20%	19.86
CPT100C30		30%	18.17
CPT150C0	150 °C	0%	41.70
CPT150C10		10%	25.83
CPT150C20		20%	18.89
CPT150C30		30%	17.29
CPT200C0	200 °C	0%	39.13
CPT200C10		10%	23.15
CPT200C20		20%	17.38
CPT200C30		30%	16.20
CPT250C0	250 °C	0%	36.65
CPT250C10		10%	21.30
CPT250C20		20%	16.25
CPT250C30		30%	14.24
CPT300C0	300 °C	0%	33.87
CPT300C10		10%	18.48
CPT300C20		20%	14.65
CPT300C30		30%	11.42

**Table 10:** The measured strains of CFRP-confined specimens.

Strain	1-Layer CFRP CPCC0-1	2-Layer CFRP CPCC0-2	3-Layer CFRP CPCC0-3
Measured strain (%)	0.89	1.09	1.22
	0.97	1.07	1.17
	1.18	1.12	1.15
Average strain (%)	0.01020	1.10	1.18

**Table 11:** The internal friction angles of the model from regression analysis.

Material Parameters	1-Layer CFRP	2-Layer CFRP	3-Layer CFRP
*f’_l_* (MPa)	7.84	15.67	23.51
Internal friction angle (*ϕ*)	27.9	20.7	12.0

**Table 12:** The *f’_c_/f’_l_* values for different perlite ratios and confined by different numbers of layers of CFRP.

-	-	Perlite Ratio in Weight			
-	No. of CFRP Layers	0%	10%	20%	30%
$\frac{f_{c}^{\prime }}{f_{l}^{\prime }}$ Value	1	5.45	2.35	1.55	0.96
	2	2.73	1.18	0.77	0.48
	3	1.82	0.78	0.52	0.32

**Table 13:** Comparison between experimental and proposed theoretical compressive peak strengths.

Specimen	Number of CFRP Layers	Average Experimental Compressive Peak Strength (MPa)	Theoretical Compressive Peak Strength (MPa)	Absolute Error (%)
CPCC0_1	1	71.76	74.78	4.67
CPCC10_1	1	42.48	40.17	5.39
CPCC20_1	1	37.73	31.90	15.43
CPCC30_1	1	29.67	25.99	12.30
CPCC0_2	2	106.18	88.26	16.72
CPCC10_2	2	55.71	56.34	1.58
CPCC20_2	2	47.51	48.32	1.72
CPCC30_2	2	43.92	42.52	4.75
CPCC0_3	3	129.32	104.04	19.55
CPCC10_3	3	79.33	72.81	8.20
CPCC20_3	3	62.58	64.86	3.73
CPCC30_3	3	49.40	59.10	19.77

Average absolute error (%).

**Table 14:** Compression test results of unconfined specimens under various temperature.

-	Δ*T* = °C	Perlite Ratio in Weight (%)			
-		0	10	20	30
Average compressive peak strength (MPa)	0	55.25	29.33	22.70	19.29
	75	47.02	26.86	19.86	18.17
	125	41.7	25.83	18.89	17.30
	175	39.13	23.15	17.38	16.2
	225	36.65	21.3	16.25	14.24
	275	33.87	18.48	14.65	11.42
Material parameter (λ)	-	0.00176	0.00164	0.00153	0.00172

**Table 15:** Absolute errors between experimental and theoretical results.

-	Δ*T* (°C)	Perlite Ratio in Weight (%)			
-		0	10	20	30
Absolute error (%)	0	0	0	0	0
	75	3.16	3.43	1.90	6.69
	125	6.51	7.51	1.64	10.01
	175	3.96	4.85	3.78	11.72
	225	2.24	4.80	1.53	7.71
	275	0.99	1.64	5.34	5.59
Average absolute error (%)	-	2.81	3.71	2.37	6.95
Correlation coefficient (*R^2^*)	-	0.96	0.97	0.98	0.92
